# Characterization of an Acidic Chitinase from Seeds of Black Soybean (*Glycine max* (L) Merr Tainan No. 3)

**DOI:** 10.1371/journal.pone.0113596

**Published:** 2014-12-01

**Authors:** Ya-Min Chang, Li-Chun Chen, Hsin-Yi Wang, Chui-Liang Chiang, Chen-Tien Chang, Yun-Chin Chung

**Affiliations:** 1 Department of Food and Nutrition, Providence University, Taichung, Republic of China (Taiwan); 2 Department of Food Science, Central Taiwan University of Science and Technology, Taichung, Republic of China (Taiwan); Russian Academy of Sciences, Institute for Biological Instrumentation, Russian Federation

## Abstract

Using 4-methylumbelliferyl-β-D-N,N′,N″-triacetylchitotrioside (4-MU-GlcNAc_3_) as a substrate, an acidic chitinase was purified from seeds of black soybean (*Glycine max* Tainan no. 3) by ammonium sulfate fractionation and three successive steps of column chromatography. The purified chitinase was a monomeric enzyme with molecular mass of 20.1 kDa and isoelectric point of 4.34. The enzyme catalyzed the hydrolysis of synthetic substrates *p*-nitrophenyl N-acetyl chitooligosaccharides with chain length from 3 to 5 (GlcNAc_n_, n = 3-5), and *p*Np-GlcNAc_4_ was the most degradable substrate. Using *p*Np-GlcNAc_4_ as a substrate, the optimal pH for the enzyme reaction was 4.0; kinetic parameters *K*_m_ and *k_cat_* were 245 µM and 10.31 min^−1^, respectively. This enzyme also showed activity toward CM-chitin-RBV, a polymer form of chitin, and N-acetyl chitooligosaccharides, an oligomer form of chitin. The smallest oligomer substrate was an N-acetylglucosamine tetramer. These results suggested that this enzyme was an endo-splitting chitinase with short substrate cleavage activity and useful for biotechnological applications, in particular for the production of N-acetyl chitooligosaccharides.

## Introduction

Chitin, an insoluble linear β-1, 4-linked polymer of N-acetylglucosamine (GlcNAc), is widely distributed in exoskeletons of arthropods, shells of mollusks, and cell walls of fungi. Apart from cellulose, chitin is the most abundant biomass in nature. Chitinases (EC 3.2.1.14) are enzymes that randomly hydrolyze β-1, 4-N-acetylglucosaminide linkage in the chitin polymer and produce bioactive N-acetyl chitooligosaccharides (GlcNAc_n_) and N-acetyl-D-glucosamine (GlcNAc). Chitinases are abundant in nature, occurring in plants, animals, viruses, bacteria, fungi and insects, and play key roles in various functions including defense, nutrient digestion, morphogenesis, and pathogenesis [1]. In plants, chitinases act as proteins for self-defense against chitin-containing fungal pathogens and insect pests [2]. During the previous decades, chitinases have increased attention because of their wide range of biotechnological applications [3].

Chitinases are classified into two glycosyl hydrolase families, namely family 18 and 19, on the basis of homology of their amino acid sequences and their catalytic mechanisms [4]. Members belonging to family 18 of chitinases are widely distributed among microbes, animals and other organisms. On the other hand, family 19 of chitinases exist mainly in higher-order plants. Plant chitinases are classified into seven classes (class Ι through VΙΙ) [5]. Most plant chitinases, however, belong to class I through IV, and each of the remaining three classes (class V-VΙΙ) currently have only one or two examples. Plant chitinases have been reported to exist in acidic and basic forms according to their isoelectric points. The acidic chitinases were transported outside the cell, and basic ones were accumulated in the vacuole [6].

The presence of chitinase in plant seeds was initially described by Powning and Irzykiewicz [7]. They found that the highest chitinase activity occurred in soybean, wheat and cabbage and proposed firstly that the enzyme served as a defense mechanism against the invasion of fungal pathogens whose cell walls contained chitinous substance. Soybean is a potential source of chitinase for use in the production of chitin degradation products. Wadsworth and Zikakis [8] partially purified a chitinase with average molecular mass of 31.6 kDa from soybean seeds, and their results suggested that the enzyme acted as an endochitinase and several isoenzymes might be present. Yeboah et al. [9] characterized a class ΙΙΙ acidic endochitinase with molecular mass of 28 kDa from soybean seeds. Northern blot analysis demonstrated that this class ΙΙΙ chitinase was specifically expressed in the developing seeds of soybean. Gijzen et al. [10] isolated a class I chitinase with molecular mass of 32 kDa from soybean seed coat and characterized its corresponding cDNA and genomic DNA. RNA gel blot analysis demonstrated that this enzyme was expressed late in seed development, with particularly high expression in the seed coat. Recently, we tested the chitinase activities in several bean seeds including soybean, black soybean, adzuki bean, mung bean and pea garden bean, and found the black soybean seeds exhibited the highest activity among tested seeds.

Chitinases are essential for the enzymatic production of GlcNAc_n_ and GlcNAc. Research regarding chitinases in various organisms will not only clarify their physiological roles but will also be of use in the production of GlcNAc_n_ and GlcNAc. In the present study, we purified and characterized an acidic chitinase with a molecular mass of 20.1 kDa from black soybean seeds. The purified enzyme hydrolyzed chitin polymer as well as chitin oligomers. Thus, we expect the chitinase from black soybean seeds can be applied in the production of oligosaccharides as biologically active substances [11].

## Materials and Methods

### Bean seeds

Black bean (*Glycine max (L)* var. Tainan no. 3) seeds, mung bean (*Vigna radiata* (L) var. Tainan no. 5) seeds and pea garden bean (*Pisum sativum* (L) var. Taichung no. 14) seeds were purchased from Tainan district agricultural research and extension station, Republic of China (Taiwan). Soybean (*Glycine max* (W) Kaohsiung no. 10) seeds and adzuki bean (*Vigna angularis* (W) var. Kaohsiung no. 7) seeds were purchased from Kaohsiung district agricultural research and extension station, Republic of China (Taiwan).

After washed and air dried, seeds were ground into fine powder (∼100 mesh), and stored at −80°C.

### Chemicals

4-Methylumbellifery-β-D-N,N,′N″-triacetyl chitotrioside (4-MU-GlcNAc_3_), 4-methylumbelliferone (MU), *p*-nitrophenyl-N-acetyl-β-D-glucosaminide (*p*Np-GlcNAc), N-acetyl-D-glucosamine (GlcNAc), N,N′-diacetylchitobiose (GlcNAc_2_), N,N′,N″-triacetyl chitotriose (GlcNAc_3_), Tetra-N-acetylchitotetraose (GlcNAc_4_), and penta-N-acetylchitopentaose (GlcNAc_5_) were purchased from Sigma-Aldrich Fine Chemicals Inc. (St. Louis, MO, USA). *p*-Nitrophenyl di-N-acetyl-β-chitobioside (*p*-Np-GlcNAc_2_), *p*-nitrophenyl tri-N-acetyl-β-chitotrioside (*p*Np-GlcNAc_3_), *p*-nitrophenyl tetra-N-acetyl-β-chitotetraoside (*p*Np-GlcNAc_4_) and *p*-nitrophenyl penta-N-acetyl chitopentaoside (*p*Np-GlcNAc_5_) were purchased from Seikagaku Corporation, Chou-Ku, Tokyo. Bicinchoninic acid protein assay reagent was obtained from Pierce (Rockford, IL, USA). Sephacryl S-100 HR, Polybuffer exchange PBE-94, Polybuffer 74, gel filtration LMW calibration kit, SDS- polyacrylamide gel electrophoresis LMW calibration kit, PhastGel Blue R, PhastGel gradient 8–25%, PhastGel IEF 3–9 and p*I* calibration kit (p*I* 3.5–9.3) were of Pharmacia (Uppsala, Sweden). CM-chitin-RBV was purchased from Loewe Biochemica GmbH (Sauerlath, Germany). Buffer salts and other chemicals used were of reagent grade.

### Preparation of crude enzyme from black soybean seeds

A 40 g of the ground black soybean powders was mixed with 400 mL of 0.1 M sodium acetate buffer (pH 4.0) and stirred for 30 min in cold room (4°C). Insoluble substances were pelleted by centrifugation (12,500 *g*, 20 min). The supernatants were collected and assayed for chitinase activity.

### Chitinase purification

Unless otherwise stated, all purification steps were carried at 4°C. Ammonium sulfate was added to the crude enzyme, and the precipitate formed upon 40% saturation was collected by centrifugation (12,500 *g*, 20 min) and dissolved in 25 mM imidazole-HCl buffer (pH 7.4). After centrifugation (12,500 *g*, 20 min), the supernatant was concentrated to a volume of 10 mL using an Amicon ultra-15 ultrafiltration unit with a 10-kDa molecular weight cutoff (MWCO) membrane (Millipore Co., Bedford, MA). The concentrated supernatant was then applied to a Sephacryl S-100 HR column (2.6×70 cm) that was pre-equilibrated with 25 mM imidazole-HCl buffer (pH 7.4). Samples were eluted with the equilibrium buffer at a flow rate of 30 mL/h. Ten-milliliter fractions were collected and their absorbance values (A_280_) as well as the chitinase activity were determined.

Fractions containing chitinase activity were pooled and concentrated to approximately 2 mL by ultrafiltration (10-kDa MW cutoff). The enzyme solution was then fractionated by isoelectric chromatofocusing on a Polybuffer exchanger (PBE-94) column (1.0×20 cm). The column was pre-equilibrated with 25 mM imidazole-HCl buffer (pH 7.4) at a flow rate of 30 mL/h. Two milliliter of the enzyme solution was applied to the 25 mM imidazole-HCl (pH 7.4) pre-equilibrated column. After sample absorption, the column was washed with the same buffer at a flow rate of 30 mL/h to elute the unbound proteins. Samples were collected every 4 mL for 6 fractions. Polybuffer 74-HCl at pH 4.0 was then applied to the column to elute the bound proteins at a flow rate of 30 mL/h. Samples were collected every 4 mL for another 70 fractions.

Fractions with chitinase activity were pooled and concentrated by ultrafiltration The concentrated enzyme solution was applied to a Sephacryl S-100 HR column (2.6×70 cm) pre-equilibrated with 25 mM imidazole-HCl buffer (pH 7.4). Samples were eluted with the equilibrium buffer at a flow rate of 30 mL/h. Five-milliliter fractions were collected and assayed for chitinase activity and absorbance values were measured at 280 nm.

### Protein determination

Protein was measured according to the bicinchoninic acid assay using bovine serum albumin as the standard [12].

### Chitinase activity determination

Chitinase activity was determined using 4-MU-GlcNAc_3_ as a substrate. A mixture of 1.0 mL of 2.5 µM 4-MU-GlcNAc_3_ in 0.05 M sodium acetate buffer (pH 5.0) and 0.02 mL of properly diluted enzyme solution in a total volume of 1.02 mL was incubated at 40°C for 30 min as described previously [13]. One unit of enzyme activity for 4-MU-GlcNAc_3_ hydrolysis was defined as the amount of enzyme releasing 1 nmole of 4- MU per minute under assay conditions.

### Molecular mass determination

The molecular mass of chitinase was estimated using gel filtration on Sephacryl S-100 HR. Bovine serum albumin (67 kDa), ovalbumin (43 kDa), chymotrypsinogen A (25 kDa) and ribonuclease A (13.7 kDa), purchased from Pharmacia Co., were used as standards. The molecular mass of the enzyme was further checked using sodium dodecyl sulfate–polyacrylamide gel electrophoresis (SDS-PAGE). SDS-PAGE was carried out with a PhastGel gradient 8–25% using the Pharmacia PhastSystem flat bed apparatus (Amersham Pharmacia Biotech AB; Uppsala, Sweden). The detail procedure is outlined in the Pharmacia PhastSystem Separation Technique File No. 110. Phosphorylase *b* (94 kDa), bovine serum albumin (67 kDa), ovalbumin (43 kDa), carbonic anhydrase (30 kDa), soybean trypsin inhibitor (20.1 kDa) and α-lactalbumin (14.4 kDa) were used as standards.

### Isoelectric point (p*I*) determination

The p*I* of the purified chitinase was measured by isoelectric focusing (IEF) on PhastGel 3–9 and compared with standards from an IEF calibration kit (Pharmacia LKB) according to the manufacturer's instructions (PhastSystem Separation Technique File No. 100.). The standard IEF conditions included carrier ampholytes prefocusing at 75 Vh and sample focusing at 410 Vh at 2.5 mA and 15°C. Sample was applied to the gel using an 8×1 µL comb. After electrophoresis, the PhastGle was stained for protein content with coomassie blue R-250. Chitinase activity in the gel was measured using the replica gel technique used by McBride et al. [14] The replica gel consisted of 10% polyacrylamide gel and 0.1% of ethylene glycol chitin. Chitinase activity was detected using Calcofluor white M2R staining [15].

### Optimal pH and temperature determination

The optimal pH for the purified chitinase for chitin oligomer substrate hydrolysis was assayed in a universal buffer (Briston and Robinson type) [16] from pH 2 to 10 at 50°C using 4-MU-GlcNAc_3_ and *p*Np*-*GlcNAc_4_ as the substrate. The optimal temperature for the purified enzyme was assayed at pH 5 (4-MU-GlcNAc_3_) and pH 4 (*p*Np*-*GlcNAc_4_) from 30 to 80°C.

### Effect of metal ions

The purified chitinase (0.3 mL, 5.57 mg/mL) was incubated with 0.3 mL of 10 mM HgCl_2_, AgNO_3_, KCl, NaCl, CaCl_2_, MgCl_2_, ZnCl_2_, MnSO_4_, or ethylenediaminetetraacetic acid (EDTA) at 25°C for 30 min. The activity for 4-MU-GlcNAc_3_ hydrolysis was then measured. The relative activity was expressed as the percentage ratio or the specific activity (units/mg) of the enzyme with metals or salts to that without metals or salts.

### Chemical modification

Aliquots of the purified chitinase (0.5 mL) were incubated with 0.5 mL of the reaction buffer corresponding to each chemical modification reagent and 0.25 mL of NBS (1, 10 mM in 50 mM acetate buffer, pH 4.0), EAM (0.5 mM in 0.2 M phosphate buffer, pH 8.0), WRK (100 mM in 0.25 M phosphate buffer, pH 6.0), NAI (5 mM in 25 mM phosphate buffer, pH 7.0) DEPC (5 mM in 0.25 M phosphate buffer, pH 6.0), *p*HMB (1 mM in 50 mM phosphate buffer, pH 7.0), CHD (5 mM in 0.1 phosphate buffer, pH 8.0), DNFB (5 mM in 0.1 M phosphate buffer, pH 8.0), PMSF (5 mM in 0.1 phosphate buffer, pH 8.0) or water as the control at 37°C for 10 min. [17], [18] Following treatment, enzyme activity in each sample was measured and expressed as a relative activity percentage calculated by the ratio of specific activity in each enzyme treated with chemical modification reagent to that of the untreated sample.

### Thin-layer chromatography (TLC) of hydrolysis products

Twenty five *µ*L of the purified enzyme was incubated with 100 µL of 5 mM GlcNAc_n_ (n = 1–5) in a thermostatic water bath at 37°C for 48 h. The enzymatic hydrolysis products were subjected to TLC on a silica gel plate 60 F_254_ (Merck), in a solvent system composed of *n*-propanol/water/ammonia water (70∶30∶1, v/v). The TLC plates were developed by first dipping them in acetone saturated with silver nitrate, followed by sprinkling with ethanol containing 0.5 N NaOH [19].

### Kinetic measurements

Two synthetic chitin oligomer substrates, 4-MU-GlcNAc_3_ and *p*Np-GlcNAc_4_, and a dye-labelled chitin polymer CM-chitin-RBV, were used to determine the substrate specificity and kinetic parameters, *K_m_* and *V*_max_ and *k_cat_*, of the purified chitinase.

The kinetics of 4-MU-GlcNAc_3_ hydrolysis were determined at pH 5.0 and 40°C for 30 min. The release of 4-MU was measured at extinction 355 nm and emission 460 nm as described above.

For the kinetics of *p*Np-GlcNAc_1-5_ hydrolysis, the reaction mixtures containing 1.0 mL of substrate in 0.05 M acetate buffer (pH 4.0) and 0.1 mL of enzyme solution in a total volume of 1.1 mL were incubated at 37°C for 30 min. The reaction was terminated by the addition of 2 mL of 0.25 M Na_2_CO_3_. The release of *p*-nitrophenol was measured by determining the absorbance at 405 nm using an extinction coefficient (ε) of 18,300 M^−1^ em^−1^ [20].

The kinetics of CM-chitin-RBV hydrolysis were determined according to Wirth and Wolf [21]. The reaction mixtures containing 0.8 mL of CM-chitin-RBV and 0.2 mL of enzyme solution in 0.1 M acetate buffer (pH 4.0) in a total volume of 1 mL were incubated at 37°C for 30 min. The reaction was terminated by the addition of 0.2 mL of 1.0 M HCl. After centrifugation (10000 *g*, 10 min), the absorbance of supernatant solution was measured at 550 nm.

The kinetic parameters, *K*_m_ and *V*_max_, were calculated from the initial rate activities of the enzyme using chitin polymer and oligomer substrates by linear regression from Lineweaver-Burk plots. Turnover number (*k_cat_*) was calculated from *V*_max._

### Data analysis

Measurements were performed in triplicate to assay the effects of various metal ions and chemical modification agents on the activity of the purified enzyme. The data were expressed as the mean ± standard deviation. Analysis of variance was performed by ANOVA using the SAS program (version 9.1, Statistical Analysis System Inc., Cary, NC; 2006). Mean values were compared by Student's t-test. A significance level of less than 5% was adopted for all comparisons. Other analytic measurement was performed in duplicate.

## Results

### Chitinase activities of bean seeds

Crude enzymes of black bean seeds, mung bean seeds, pea garden bean seeds, soybean seeds and adzuki bean seeds were prepared with 0.1 M sodium acetate buffer (pH 4.0), and their chitinase activity were 11.13, 0.35, 0.012, 5.58 and 0.167 unit/g, respectively, toward 4-MU-GlcNAc_3_. Black bean seeds exhibited the highest chitinase activity among test seeds, therefore, the chitinase of black bean seed was purified and its biochemical properties were evaluated.

### Chitinase purification

Chitinase was purified to homogeneity from black soybean seeds following the procedures described in section 2.4. Chitinase was first precipitated from the crude extract using 40% saturation of ammonium sulfate and further purified through three successive column chromatography steps. The protein elution profile obtained at the first gel filtration step showed that a major protein peak with chitinase activity was separated from three protein peaks without chitinase activity (Data not shown). The protein elution profile obtained at second chromatofocusing step showed that a lot of proteins without chitinase activity were eluted at pH 7.4 to 4.5 whereas chitinase was eluted at pH 4.0 (Data not shown). The final gel filtration step on Sephacryls-100 HR showed that a protein peak with chitinase activity was separated from a small Polybuffer peak with absorbance at 280 nm (data not shown). The result of the purification procedure is summarized in Table 1. The purity of the enzyme was estimated to be about 10.7 fold greater than that of crude extract with a yield of 8.2%.

**Table 1:** Purification of chitinase from black soybean seeds.

Procedure	Total volume (mL)	Total protein (mg)	Total activity b (units)	Specific activity (units/mg)×100	Purification (fold)	Yield (%)
**Crude enzyme a**	340	1607	146	9.1	1	100
(40% saturation) (NH_4_)_2_SO_4_	23	650	133	20.5	2.6	91.1
Sephacryl S-100 HR gel filtration	100	245	70	28.7	3.1	47.9
PBE-94 chromatofocusing	113	43.5	35.1	80.7	8.9	24.0
Sephacryl S-100 HR gel filtration	33	12.3	12.0	97.6	10.7	8.2

a Data were obtained from forty gram of black soybean seeds.

b Chitinase activity was determined using 4-MU-GlcNAc_3_ as the substrate. One unit of enzyme activity was defined as the amount of enzyme releasing 1 nmole of 4-methylumbelliferone (MU) per minute under assay conditions.

### Molecular mass determination

**Figure 1:**
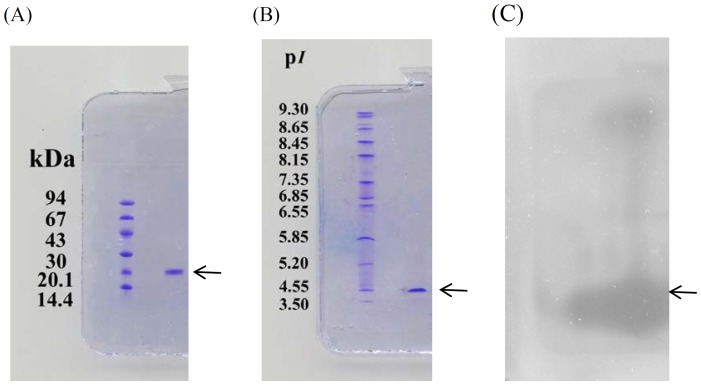
SDS-PAGE (A) and IEF-PAGE (B and C) of the purified chitinase from black soybean seeds. SDS-PAGE was performed in PhastGel 8–25% with PhastGel SDS buffer strips. Lanes 1 indicates purified black-soybean chitinase; Lane S indicates low molecular weight standard proteins ranged from 14.4 to 94 kDa. IEF-PAGE was performed on PhastGel IEF 3–9 gels containing wide-range ampholytes (p*I* 3–10). Lane S contains p*I* marker proteins. Lane 1 contains purified chitinase. Protein detected by coomassie blue R-250 staining (B). Chitinase activity detected using glycol chitin overlay (C).

The homogeneity of the purified chitinase was examined by SDS-PAGE under reducing conditions and by protein staining analysis, and a unique protein band appeared on SDS-PAGE (Figure. 1A). The purified chitinase had a molecular mass of 20.1 kDa which was corresponding to the result of gel filtration on Sephacryl S-100 HR (data not shown).

### Isoelectric point

The isoelectric point (p*I*) of the purified enzyme was analyzed by IFF electrophoresis and activity staining. By protein staining, a unique protein band with isoelectric point of 4.34 was obtained (Figure 1B). By activity staining, a major activity band, which matched the protein band, and a minor activity band with basic isoelectric point were obtained (Figure 1C). Thus the isoelectric point of the purified enzyme was 4.34.

### Optimal pH and temperature for enzyme reaction

The optimal pH of purified chitinase for hydrolyzing 4-MU-GlcNAc_3_ and *p*Np-GlcNAc_4_ were pH 5 and 4, respectively; whereas the optimal temperatures were 40 and 50°C, respectively (data not shown).

### Substrate specificity and kinetic properties

**Figure 2:**
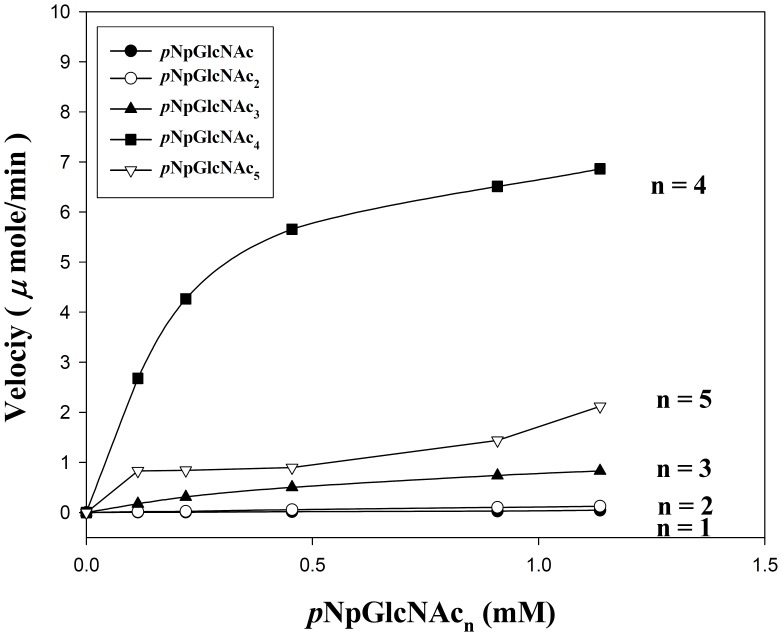
Effect of concentration of synthetic substrates with chain length from 1 to 5 on the activity of the purified chitinase from black soybean seeds.

Figure 2 presented the effect of substrate concentration on the rate of *p*Np-GlcNAc_n_ (n = 1–5) hydrolysis and showed that the most digestible *p*-nitrophenyl N-acetyl chitooligosaccharides was *p*Np-GlcNAc_4_. When 4-MU-GlcNAc_3_ was used as a synthetic substrate, the enzyme showed a *K*_m_ value of 17.5 µM and a value of *k*_cat_ 0.338 min^−1^. When *p*Np-GlcNAc_4_ was used as a synthetic substrate, the enzyme showed a *K*_m_ value of 245 µM and a *k*_cat_ value of 10.31 min^−1^ (Table 2).

**Table 2:** Kinetic parameters of purified chitinase from black soybean seeds for a chitin polymer derivative CM-chitin-RBV and two synthetic substrates.

Substrate	*k*_cat_ (min^−1^)	*V*_max_ (A_550_ min^−1^mg^−1^)	*K*_m_ (*µ*M)	*K*_m_ (mg/mL)	*k*_cat_/*K*_m_ (min^−1^. *µ*M^−1^)
4-MU-GlcNAc_3_	0.338	**—**	17.45	**—**	19.37×10^−3^
*p*Np-GlcNAc_4_	10.31	**—**	245	**—**	42.01×10^−3^
CM-chitin-RBV	**—**	19.61	**—**	2.48	**—**

The purified chitinase had much lower *K*_m_ value for 4-MU-GlcNAc_3_ than for *p*Np-GlcNAc_4_; however, the enzyme exhibited much higher catalytic velocity (*k*_cat_) and catalytic efficiency parameter (*k*_cat_/*K*_m_) for *p*Np-GlcNAc_4_ than for 4-MU-GlcNAc_3_.

On the other hand, the purified chitinase was capable of hydrolyzing CM-chitin-RBV, a water soluble-chitin derivative, with a *K*_m_ value of 2.48 mg/mL^−1^ and a *V*_max_ value of 19.61 A_550_ min^−1^mg^−1^ (Table 2).

### Hydrolytic products from chitin oligosaccharides

**Figure 3:**
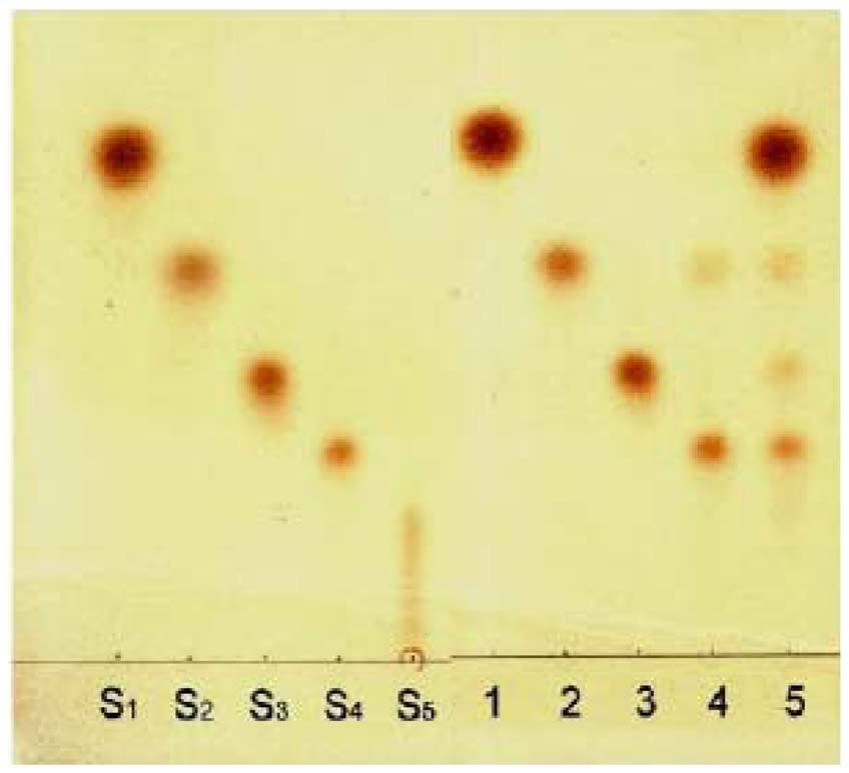
Thin-layer chromatogram showing hydrolysis of chitin oligomers (GlcNAc_2–5_) by the purified chitinase from black soybean seeds. Lanes S_1_ through S_5_ indicate standard N-acetylglucosamine monomer and oligomers GlcNAc, GlcNAc_2_, GlcNAc_3_, GlcNAc_4_ and GlcNAc_5,_ respectively. Lanes 1 through 5 indicate hydrolysis products of N-acetylglucosamine oligomers from monomer to pentamer respectively.

The products of chitin oligomer hydrolyzed by the purified chitinase were determined by TLC. As shown in Figure 3 the purified chitinase did not cleave chitin dimer and trimer. It partially cleaved chitin tetramer into the dimer (GlcNAc_2_) and completely cleaved chitin pentamer into a mixture of monomer (GlcNAc), dimer (GlcNAc_2_), trimmer (GlcNAc_3_) and tetramer (GlcNAc_4_). The results mentioned above suggesting that the enzyme acted as an endo-splitting enzyme.

### Effectors and inhibitors

Table 3 showed the effects of metal ions and chemical modification reagents on the chitinase activity. When the purified chitinase was incubated with various group-specific reagents for amino acid modification, its activity was noted to be almost completely inhibited in the presence of 5 mM of NBS. Purified chitinase lost partially its activity when it was incubated with EAM, NAI, DNFB, DEPC, WRK or *p*HMB. PMSF did not affect the activity; however, the chitinase activity was increased about 26% with addition of CHD.

**Table 3:** Effects of various metal ions and chemical modification reagents on the activities of chitinase from black soybean seeds.

Metal ions	Final concentration (mM)	Relative activity x (%)	Chemical modificationreagent	Final concentration (mM)	Relative activity (%)
None	**—**	100	None (control)	**—**	100
HgCl_2_	0.25	83.5±1.6 **	NBS	5.0	5.09±2.3 **
AgNO_3_	0.25	86.4±2.3 **	NBS	0.5	78.96±1.5 **
KCl	5.0	92.8±1.2 *	EAM	0.25	65.13±3.1 **
NaCl	5.0	95.0±3.7	NAI	2.5	67.74±2.0 **
CaCl_2_	5.0	95.2±4.2	CHD	2.5	126.46±5.5 **
MgCl_2_	5.0	103.5±2.8	DNFB	2.5	78.18±1.9 **
ZnCl_2_	5.0	96.4±3.5	PMSF	2.5	104.33±4.1
MnSO_4_	5.0	93.2±4.1 *	DEPC	2.5	75.96±2.4 **
EDTA y	5.0	90.0±0.8 **	WRK	50	83.28±0.9 **
			PHMB	0.5	71.01±1.7 **

Values were means ±S.D (n = 3).

x Enzyme activity was determined using 4-MU-GlcNAc_3_ as the substrate.

y EDTA: ethylenediamine tetraacetic acid disodium salt.

*Significant difference between the control and treated groups at *p*<0.05.

**Significant difference between the control and treated groups at *p*<0.01.

Since NBS usually acts on Trp and Cys; EAM and DNFB usually act on Lys; NAI, DEPC, PHMB and WRK are specific modification reagents for Tyr, His, Cys and acidic amino acids, respectively. Therefore, Trp, Lys, Tyr, His, Asp, Glu, and Cys residues are probably involved in the enzyme catalytic reaction. When the enzyme was incubated with various metal ions, its activity was partially inhibited by Hg^2+^, Ag^+^, Mn^2+^ and EDTA but no significant effect in the presence of other metal ions.

## Discussion

In this work, using 4-MU-GlcNAc_3_ as a fluorogenic synthetic substrate, we purified a chitinase from black soybean seeds. The purified enzyme exhibited a unique protein on SDS-PAGE under reducing conditions. The homogeneity of the purified chitinase was also checked by isoelectric focusing electrophoresis and activity staining on PhastGel IEF 3–9. A unique protein band with isoelectric point of 4.34 was obtained for the purified enzyme. Zymogram analysis indicated that the unique protein band with isoelectric of 3.34 exhibited chitinase activity and that trace amount of basic chitinase isoform was co-purified in the enzyme preparation. The purified enzyme had a molecular mass of 20.1 kDa, as estimated by SDS-PAGE, and this value was close to that estimated by gel filtration on Sephacryl S-100 HR. These results suggested that the purified enzyme is a monomeric acidic chitnase. Plant chitinases typically exist as monomers and their molecular mass commonly range from 25 to 35 kDa. They may be acidic or basic and may contain a putative chitin-binding domain in addition to the catalytic domain [22]. However, the values of molecular mass of chitinase reported for carrot (12.5–20.5 kDa) [23], Azuki bean (21.0 kDa) [24] and seeds of *Zea mays* (11.5 kDa) and *Coix lachruma-jobi* L. (10.0 kDa) [25] are smaller than most of plant chitinases.

Many research reported that soybean seeds contained several isoenzymes of chitinases and all purified chitinase isolated from soybeans seeds showed molecular mass greater than 30 kDa. For example, Wadsworth and Zikakis [8] applied chitin chromatography to elute a variable number of chitinases with higher molecular mass, in which, proteins with molecular weight of 82–94.5 kDa and 43–62 kDa were predominant. In Wadsworth and Zikakis' work [8], a group of chitinases, with average molecular mass of 31.6 kDa, was partially purified and those enzymes degraded chitobiose as well as microcrystalline chitin. Yeboah et al. [9] purified a chitinase with molecular mass of 28 kDa from soybean seeds, and their work demonstrated this enzyme belonged to the class ΙΙΙ acidic endochitinase toward ethylene glycol chitin. Gizen et al. [10] obtained a class I chitinase with molecular mass of 32 kDa from soybean seed coat, and this enzyme degraded glycol chitin. According to the literatures mentioned above, isolated chitinases had molecular mass close to 30 kDa and showed major hydrolysis activities toward polymer form of chitin. In this study, we also found chitinase isoenzymes in black bean seeds with molecular mass range of 20–30 kDa, and we purified the smaller protein only because the 20 kDa protein was not much of discussed; besides, this 20 kDa protein degraded both polymer and oligomer form of chitin. Compared to published data, the chitinase obtained in this study not only had smaller molecular size, also preferred smaller substrates.

Using CM-chitin-RBV as a substrate for activity assessment of the purified enzyme, the liberated products of dye-labelled oligomers from CM-chitin-RBV were detected in the supernatant of reaction mixture by the addition of HCl. The liberated oligomer fragments with dye-labelling were acid-soluble whereas the remaining polymer fragments were acid-insoluble. These results indicated that the enzyme degraded chitin polymer substrate.

Using chitin oligomers as substrates, chitin oligomers GlcNAc_2_ and GlcNAc_3_ were not cleavable. Accordingly, this enzyme did not show exo-type chitinolytic activity. However chitin oligomers GlcNAc_4_ and GlcNAc_5_ were cleaved by the enzyme. GlcNAc_4_ was partially cleaved in a specific way to two molecules of GlcNAc_2_. GlcNAc_5_ was completely cleaved into GlcNAc, GlcNAc_2_, GlcNAc_3_ plus GlcNAc_4_ (Figure. 3). The GlcNAc_5_ might be cleaved at the fourth glycosidic linkage from the non-reducing end to GlcNAc plus GlcNAc_4_ and then the resulting GlcNAc_4_ was cleaved further to two molecules of GlcNAc_2_. It might be also possible that the GlcNAc_5_ was cleaved at the second or third glycosidic linkage from the non-reducing end to produce GlcNAc_2_ plus GlcNAc_3_. Rice chitinase isoforms OsChib 1a and OsChib 1b were assumed to have similar sugar-binding sub-sites (−4 to +2) assigned for glycosyl hydrolases [26], [27]. Experiment with GlcNAc_n_ (n = 4–6) showed that OsChib 1a efficiently hydrolyzed GlcNAc_4_ and GlcNAc_5_ as well as GlcNAc_6_. GlcNAc_4_ appeared to bind this enzyme exclusively at the sub-sites −2 to +2, leading to symmetrical cleavage of GlcNAc_4_ between -1 and +1 to produce two molecules of GlcNAc_2_. On the other hands, GlcNAc_5_ would bind to the sub-sites through −4 to +2 in all possible ways, thus resulting in the formation of a variety of products.

Kinetic experiments revealed that the purified enzyme did not show any activity toward *p*Np-GlcNAc and *p*Np-GlcNAc_2_. However, the enzyme hydrolyzed the synthetic substrates with chain length from 3 to 5. The most effective hydrolyzing substrate was *p*Np-GlcNAc_4_, followed by *p*Np-GlcNAc_5_ and *p*Np-GlcNAc_3_, respectively (Figure. 3). Since *p*-nitrophenol was released effectively from the synthetic substrates *p*Np-GlcNAc_4_, we deduced that *p*-nitrophenol moiety was bond to the binding site adjacent to the reactive site. These results suggested that this enzyme, in the same was as the fig chitinase [20], preferentially hydrolyzed the fourth glycosidic linkage from the non-reducing end of N-acetyl chitooligosaccharides.

A fluorogenic synthetic substrate 4-MU-GlcNAc_3_ and a chromogenic synthetic substrate *p*Np-GlcNAc_4_ were used to determine the kinetic parameters for the purified chitinase. The enzyme showed much higher reaction velocity (*k*_cat_) and catalytic efficiency parameters (*k*_cat_/k_m_) toward *p*Np-GlcNAc_4_ than toward 4-MU- GlcNAc_3_. The *k*_cat_ of this enzyme toward *p*Np-GlcNAc_4_ was 10.31 min^−1^. This value was within the range of *k*_cat_ values (4.2–52.8 min^−1^) of yam chitinase isoforms toward GlcNAc_3–6_ [28] but smaller than the *k*_cat_ value of barley chitinase (35 min^−1^) toward GlcNAc_4_ [29].

It is generally believed that an enzyme will be inhibited or inactivated if its amino acid side chains which are involved in catalytic activity are chemically modified. In this study, a number of reagents were used to chemically modify the amino acid side chains of the purified enzyme. The results suggested that Trp, Lys, Tyr, His, Asp, Glu and Cys residues might be involved in the substrate binding or catalytic reaction of the enzyme. So far, not much information has been reported regarding the inhibitory effect of chemical modification on plant chitinases. Fig chitinase was inhibited by NBS, CHD and DNFB and the results indicated that Trp, Lys and Arg might be related to the reaction of the enzyme [30]. A chitinase isolated from *Zea mays* seeds was inactivated by 1-ethyl-3-(3-dimethylaminopropyl) carbodiimide (EDC) and the results suggested that Tyr was essential for the catalytic reaction of enzyme [31].

## Conclusions

The present study reports a new chitinase purified from black soybean seeds. Biochemical characterization and kinetic analysis indicated that the purified enzyme was an acidic endo-splitting enzyme. The enzyme processed hydrolytic activities toward chitin polymers and oligomers. It is a potential chitinase for biotechnological applications especially for the production of biological active N-acetyl chitooligosaccharides. To open up applications and elucidate molecular structure of this chitinase, further studies are needed to reveal the mechanisms of substrate-binding and catalysis, amino acids sequence and antifugal activity.
